# Family-Focused Nursing Research in WHO Afro-Region Member States: A Scoping Review

**DOI:** 10.1177/10748407221132018

**Published:** 2022-11-26

**Authors:** Geldine Chironda, Mary Ann Jarvis, Petra Brysiewicz

**Affiliations:** 1 University of KwaZulu-Natal, Durban, South Africa

**Keywords:** Africa, family, family nursing, family nursing research, scoping review

## Abstract

Although family nursing research has become an important focus for over the past 20 years, the evolution and extent of family nursing research in the World Health Organization (WHO) Afro-regions is less explored. The purpose of this scoping review was to map the evidence of family-focused nursing research using the Joanna Briggs Institute Scoping Review methodology. A systematic electronic search of articles was carried out for the period January 1, 2000 to December 31, 2020. The review process culminated in 85 articles, evidencing an increase in publications particularly in 2019 (*n* = 12). Eighteen countries were involved, with the Southern African region contributing 52% of the studies. Family members were predominantly described as parents, siblings, and children, with the most focused area of study being family experiences (*n* = 52). The majority of studies (*n* = 59) used qualitative methodologies. Despite the recent increase in family-focused nursing research in the WHO Afro-regions, further qualitative research, including more complex methodologies and interventions are still required to build contextualized evidence-based family-focused nursing.

Societal norms and values of many African nations are rooted in the philosophy of *Ubuntu* that holds central that a person is a person because of other people; encompassing respect for humanity, compassion, prioritizing the interests of the most vulnerable, and including community solidarity (De Beer & Brysiewicz, 2016; Radebe & Phooko, 2017). Such an ideology highlights the importance of the community and family in the African context. This is particularly relevant in health care settings where the family often has to assume a role of providing care to their ill family member (Muliira & Kizza, 2019). Unfortunately, in the African health care setting, the concept of family-focused nursing is not well developed.

Globally, families have undergone structural changes due to poverty, migration, war and environmental factors, leading to their altered functioning and well-being (Castelli, 2018; Makiwane et al., 2017). The current global context of rapid technological, social, and economic changes (Ikamari & Agwanda, 2020; Luttik et al., 2020; Russell, 2020) has influenced how nurses and other health care professionals engage with families. Moreover, diseases like Covid-19 have resulted in psychological stress, social isolation, and disruptions in family life processes and functioning (Feinberg et al., 2022; Lebow, 2020). The family, conceptualized as “a group of individuals bound by strong emotional ties, a sense of belonging, and a passion for being involved in one another’s lives” (Wright & Bell, 2021, p. 61), need to overcome these challenges in attempting to care for their loved ones (Deatrick, 2017; Muzondo, 2021). This review adopts the definition of family as a system bound by biological (genetic), legal (adoption, guardianship and marriage), and sociological (friends and neighbors) ties (Erlingsson & Brysiewicz, 2015; Makiwane & Kaunda, 2018).

In light of the significance of the family, in settings where the family remains under-recognized, a shift is required to expand nursing care from being predominantly patient focused to include both the patient and their family (Østergaard & Wagner, 2014). Family-focused nursing becomes fundamental in integrating nursing care with the family as a whole and individually in health and illness contexts (International Family Nursing Association [IFNA], 2013) as positive outcomes are associated with nurse-family engagement during patients’ health care episodes. These outcomes include; fewer diagnostic tests and adverse events, decreased use of health services, shorter length of hospital stay, overall improvement in health literacy, and improved self-care in chronic disease management, clinical decision-making, and patient safety (Goodridge et al., 2018; Jazieh et al., 2018). However, the implementation and sustainability of effectively providing family-focused care in health care settings is complex and continues to be a global challenge for family-focused nurses (Duhamel, 2017). In addition, the current Covid-19 pandemic has challenged the maintenance of family relationships in health care contexts. Regulations of social isolation and distancing have meant minimal family interaction and engagement (Jarvis et al., 2021), with adverse effects (Montauk & Kuhl, 2020).

The1950s witnessed the start of nursing research about family phenomena (Feetham, 1984), with an increase in the 1980s leading to the first International Family Nursing Conference in 1988 in Calgary, Alberta, Canada; the launch of the *Journal of Family Nursing* (SAGE Publications) in 1995 (Hanson, 2005); and the establishment of the IFNA in 2009. For more than 30 years, family nursing researchers have been building a knowledge base of families’ experiences of health and illness, including family-focused assessment and intervention (Bell, 2017; Duhamel, 2017). As current changes in family life continue to unfold, so too emerges the need for ongoing innovation in methods and strategies when conducting family nursing research (Russell, 2020). However, in Africa, this is a challenge as family nursing practice is not recognized as a nursing specialty, resulting in a lack of a common definition of family and family-focused nursing, including no formal integration of family constructs into nurse education programs (Imanipour & Kiwanuka, 2020; Irinoye et al., 2006).

The extent of research studies in this area emanating from Africa is not clear. However, it is suggested that many aspects of family-focused nursing research in Africa still need to be explored; thus, the need to coalesce and synthesize the existing body of evidence. In this regard, a scoping review was deemed necessary to map and examine available literature on family nursing research in the WHO Afro-region member states by answering the following five questions: (a) What are the publication trends and the distribution of family nursing research? (b) What type of designs (methodology) has been used to explore family nursing research? (c) Which family member(s) was involved? (d) What are the focus conditions? (e) What is the family nursing research focus?

## Methods

The Joanna Briggs Institute (JBI) Scoping Review Methodology (Khalil et al., 2020), dictated the development of the review protocol, that was registered on June 26, 2020 with the Open Science Framework (https://osf.io/j972b). The Preferred Reporting Items for Systematic Reviews and Meta-Analyses extension for Scoping Reviews’ (PRISMA-ScR) checklist guided the reporting of this scoping review (Tricco et al., 2018).

### Eligibility Criteria

The JBI framework, including Population, Concept, Context (PCC) defined key inclusion and exclusion criteria (see Table 1) (Khalil et al., 2020). In addition, articles published from 2000 to 2020, in English, and authored or co-authored by a nurse determined eligibility. Published articles were limited to English as it is the main medium of publication in the WHO Afro-region member states (Plonski et al., 2013), and this also matched the team’s language skills. The use of gray literature in this scoping review was limited to completed dissertations and theses to increase the review’s comprehensiveness of available evidence (Paez, 2017). This is essential as in Africa completed dissertations or theses are not always converted into publications.

**Table 1.** Population, Concept, Context (PCC), Inclusion, and Exclusion Criteria.

PCC and type of evidence inclusion	Defining characteristics
Population	Family caregivers, caregivers, primary caregivers, female caregivers, informal caregivers, parents, spouses, siblings, bereaved caregivers, families, relatives, grandmothers, grandparents, next of kin, significant others
Concept	Caregiving, family caring, family-centered, family collaboration, family concepts, family engagement, family experiences, family-focused, family needs, family nursing, family oriented, family research, family systems, family nursing research, nursing, and midwifery
Context	The 46 countries listed within the WHO Afro-region member states (sub-Saharan Africa, Southern Africa, Central Africa, West Africa, East Africa, Mauritius, Seychelles, and Madagascar), in the context of any nursing health system or health care setting, such as in hospital, clinics primary health care, community and primary health care
Type of evidence	Quantitative, qualitative, and mixed-methods study designs that addressed family phenomena/family-focused nursing in the 46 WHO Afro-region member states Review articles including but not limited to systematic, meta-analysis, narrative, integrative and scoping reviews Gray literature sources including academic outputs (theses and dissertations)
Exclusion criteria	Defining characteristics
Reasons for exclusions	Publications before 2000 Articles written in languages other than English Articles from the WHO region of the Americas, South-East Asia region, European region, Eastern Mediterranean region and Western Pacific region Articles written by health care professionals which do not include a nurse as an author Articles with heterogeneous population where family members contribute less than 50% of the sample size Articles with multicountry collaboration research where WHO Afro-region countries are less than 50% of the included countries Review protocols labeled as incomplete research

*Note.* WHO = World Health Organization.

### Search Terms

Table 2 presents the search terms used to identify published and unpublished articles from a number of electronic data bases and search engines for the scoping review.

**Table 2.** Medical Subject Headings (MeSH) and Search Terms.

Types	Search terms
Medical subject headings terms	Family, family nursing, family research, family nursing research, Africa
Combination of search terms	((Family or primary caregivers or female caregivers, or informal caregivers, or parents, or spouses, or siblings, or relatives, grandmothers, or grandparents, or next of kin, or significant others) OR (family-oriented family needs, family systems, family-focused, family engagement, family centered, family caregivers, family caring, family experiences, family concepts))) AND ((nursing and research or midwifery and research))) AND (OR (Africa, WHO Afro-region, sub-Saharan Africa, Southern Africa, Central Africa, West Africa, East Africa, Mauritius, Seychelles and Madagascar))) AND (January 1, 2000 to December 31, 2020)

*Note.* WHO = World Health Organization.

### Search Strategy

Using the identified search terms in Table 2, a three-stage search strategy was followed. The Boolean operator “AND” narrowed the search into more focused and productive results for nursing, midwifery and research, while the operator “OR” broadened the search for the PCC. Relevant wild cards and truncations accounted for spelling and plural variations in the different databases and search engines. The initial preliminary search was conducted with two databases (PubMed and CINAHL) and using the title and abstract of retrieved articles and their index terms, discussion was held with all three reviewers to further refine the search terms.

The second search was conducted from March 26 to March 31, 2021 using all identified keywords and index terms and included the databases: AJOL, CINAHL, MEDLINE, PubMed, Sabinet, Scopus, and Web of Science, the metadata base EBSCO host, and the search engines Google and Google Scholar. Furthermore, a search of gray literature of completed unpublished academic outputs (theses and dissertations) discussing family nursing research in the WHO Afro-region was carried out by initially searching the ProQuest Dissertation and Theses Global (PQDT), then search engines Google and Google Scholar. Finally, subject area experts were consulted regarding research being conducted, as well as theses and dissertations focused on family nursing.

The third step of the search strategy involved hand searching the reference lists of the 85 identified articles for additional sources that included published and gray literature. All searches were saved in the reference manager, EndNote, and exported into the Systematic Reviews Web App (Beta) Rayyan (Ouzzani et al., 2016). An expert librarian was consulted to assist with all stages of the search strategy as well as for assistance in sourcing articles for full-text screening (McGowan et al., 2016).

### Source of Evidence Selection

All duplicates were removed (*n* = 331) and recommendations made by subject area experts allowed for the identification of further articles (*n* = 7). At the first-level screening, two of the reviewers (GC and MAJ) using Rayyan (Ouzzani et al., 2016), screened all the titles and abstracts obtained from the search (*n* = 249) against the eligibility criteria. Piloting the selection process involved blinding for the first 15 articles with the same two reviewers (GC and MAJ).

The level of disagreement was high with discrepancies emanating from an unclear Population and Context; however, this was resolved in consultation with the third reviewer (PB). The definition of family was further clarified leading to a redefining of the population to include informal caregivers, relatives, and significant others, with the exclusion of formal (paid) caregivers. The context of the WHO Afro-region states was further clarified to include the North, East, South, and West African countries as well as the islands of Seychelles, Madagascar, Mauritius, and Comoros.

**Figure 1.**
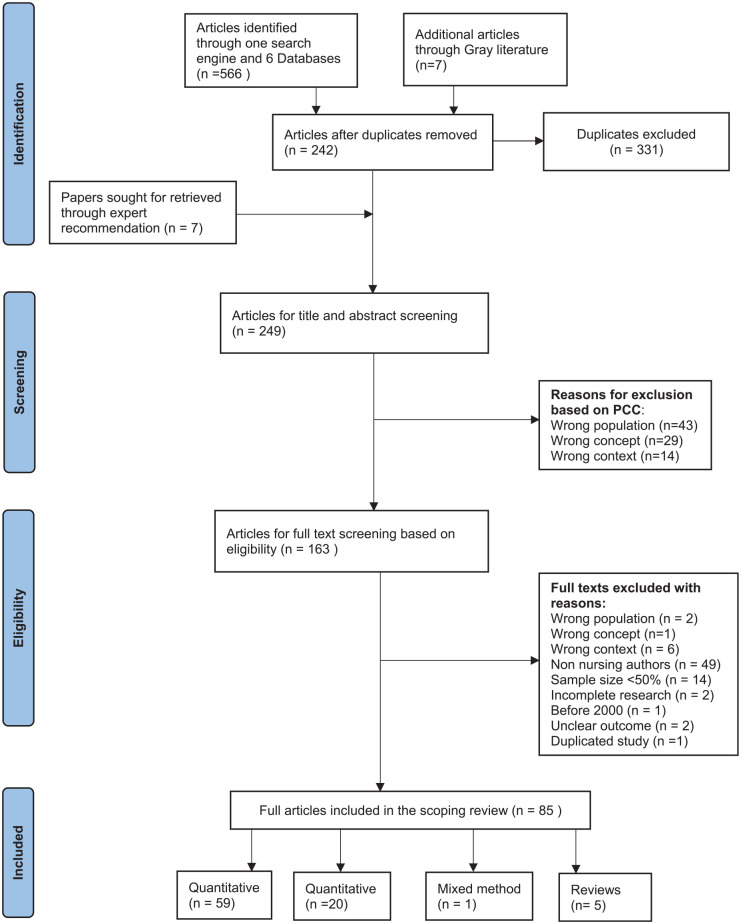
PRISMA-ScR Flow Chart for Selection of Articles on Family Nursing

Subsequently through blinding, the reviewers (GC and MAJ) independently continued with the selection process, followed by a discussion where disagreements, mainly concerning the concept of caregiving, resulted in its inclusion in the PCC. The second-level screening of full-text articles (*n* = 163) was carried out independently by two reviewers (GC and MAJ) and the final articles for inclusion were selected (*n* = 85) (Figure 1). The third reviewer (PB) reviewed and verified all the selected articles.

### Data Extraction Process

The team developed a charting table to record the key information extracted from the sources, namely: WHO Afro-region, country of study, author(s), year of publication, research design, setting, sample size, sampling strategy, family member(s) involved, and focus condition (Table 3). Charting of data was revised and modified to meet the review questions until all three reviewers reached a consensus.

**Table 3.** Synthesized Evidence of Family Nursing Articles According to WHO Afro-Region Member States (n = 85).

Country of study	Author, year of publication	Research design	Setting	Sample (*n*) /**s**ampling	Family member**(**s**)** involved	Focus condition
Central African region (**s**ingle**-**country studies) (***n*** = 2, 2.4%)						
Democratic Republic of Congo**(***n* = 2, 2.4**%)**	Nkosi et al. (2006)	Qualitative descriptive	Home-based care	12/purposive	Spouses	HIV/AIDS
	Matua & Van der Wal (2015)	Phenomenology	Community	7/purposive, convenience and criterion	Spouse, adult children and relatives	Ebola
East African region (single-country studies) (*n* = 22, 25.9**%)**						
Ethiopia (*n* = 4, 4.7**%)**	Biru et al. (2015)	Phenomenology	PHC facility	21/purposive	Parents, grandfathers, and relatives	HIV/AIDS
	Biru et al. (2017)	Prospective cohort	PHC facility	306/purposive	Parents, legal guardians, sibling, and extended family member	HIV/AIDS
	Biru et al. (2018)	Phenomenology	PHC facility	18/purposive	Parents and relatives	HIV/AIDS
	Aga et al. (2009)	Ethnography	Home-based care	12/Purposive	Mother, spouse, daughter, sibling, and friend	HIV/AIDS
**Kenya **(***n* = 2, 2.4**%)**	Okumu et al. (2017)	Descriptive cross-sectional survey	Hospital, pediatric oncology ward	62/purposive	Parents	Cancer
	Mugoya et al. (2020)	Descriptive cross-sectional survey	Disability health care	247/not stated	Parents, spouses, and relatives	Disabilities
Malawi **(***n* = 4, 4.7**%)**	Kululanga et al. (2012)	Qualitative exploratory descriptive	Hospital, labor ward	20/purposive	Fathers	Birth of child
	Chorwe- Sungani et al. (2015)	Qualitative descriptive	Hospital, mental health ward	10/purposive	Immediate family	Mental illness
	Gondwe et al. (2017)	Qualitative descriptive	Hospital, pediatric ward	12/purposive	Mothers	Respiratory distress
	Phiri et al. (2020)	Qualitative descriptive	Hospital, pediatric ward	20/not stated	Parents	Conditions requiring medical or surgical interventions
Mozambique **(***n* = 1, 1.2**%)**	Söderbäck & Christensson (2008)	Cross-sectional survey	Hospital, pediatric ward	100/random sampling	Parents, grandmothers and sibling	Conditions requiring medical, surgical, or emergency interventions
Rwanda **(***n* = 3, 3.5**%)**	Munyiginya and Brysiewicz (2014)	Quantitative exploratory	Hospital, ICU	40/convenience	Blood relative and significant other	Critical illness
	Musabirema et al. (2015)	Descriptive survey	Hospital, NICU	110/census	Parents	Critical illness
	Nkuranyabahiz (2019) Nkuranyabahizi et al. (2021)	Phenomenology	Hospital, renal unit	12/purposive	Parents, siblings, adult child, spouse, and friend	End-stage renal disease
Tanzania **(*n* = 4, 4.7%)**	Pallangyo & Mayers (2009)	Qualitative descriptive	PHC facility	8/purposive	Siblings, neighbors, children, and partners	HIV/AIDS
	Outwater et al. (2012)	Ethnography	Hospital, mortuary	30/convenience	Father, uncle, stepfather, cousin, sibling, and friend	Homicide
	Iseselo et al. (2016)	Qualitative descriptive	Hospital, OPD of mental health and substance abuse	14/purposive	Parents and children	Mental illness
	Shimpuku et al. (2018)	Quasi experimental	PHC facility	96/purposive	Spouses, children, relatives, neighbors, and significant others	Antenatal care
Uganda **(***n* = 4, 4.7**%)**	Olwit et al. (2015)	Qualitative descriptive	Hospital, mental health ward	8/purposive	Parent, sibling, and extended family member	Mental illness
	Olwit et al. (2018)	Qualitative descriptive	Hospital, sickle aaaaa clinic	12/purposive	Parents and guardians	Sickle aaaaa disease
	Matovu et al. (2019)	Grounded theory	Community	32/purposive	Grandparents	HIV/AIDS
	Muliira & Kizza (2019)	Cross-sectional survey	Hospital, OPD, wards and home-based care	284/not stated	Spouse, child, neighbor, relatives, and friends	Cancer
Southern African region (single-country studies) (*n*=44, 51.8**%)**						
**Botswana (*n* = 7, 8.2**%)**	Phaladze (2001)	Narrative review	Desk top review	Not applicable	Mothers, grandmothers, and sibling	HIV/AIDS
	Lindsey et al. (2003)	Qualitative descriptive	Home-based care	35/convenience	Mothers, spouse, grandmothers, sibling, uncle, and daughter in law	Chronic illnesses
	Seloilwe (2006)	Grounded theory	Hospital, mental health ward	30/convenience	Spouses, adult children, grandmothers, and relatives	Mental illness
	Letlola-Motana (2007)	Phenomenology	Hospital, NICU	8/convenience	Mothers	Critical illness
	Ncube (2011)	Phenomenology	Hospital, neonatal ward	8/purposive	Mothers	Prematurity complications
	Philips & Lazenby (2013)	Qualitative descriptive	Hospital, hospice	28/not stated	Parents, spouse, adult child, sibling, and significant others	End of life/dying
	Shaibu (2013)	Qualitative descriptive	Home-based care	12/purposive	Grandmothers	Orphans
**Namibia **(***n* = 4, 4.7**%)**	Leuning et al. (2000)	Ethnography	Home-based care	11/purposive	Grandchildren and adult children	Elderly
	Ntswane & Van Rhyn (2007)	Phenomenology	Community	12/purposive	Mothers	Intellectual disability
	Amakali & Small (2013)	Phenomenology	Rural community	5/purposive	Parents	Heart disease
	Hamukwaya (2019)	Cross-sectional survey	Hospital, ICU	130/simple random sampling	Parent, spouse, children, siblings, and grandchildren	Critical illness
**South Africa **(***n* = 32, 37.6**%)**	Davhana-Maselesele (2005)	Phenomenology	Community	purposive	Spouses	Death
	Ramnath (2007)	Cross-sectional survey	Hospital, ICU	44/convenience	Friends	Critical illness
	Brysiewicz (2008)	Phenomenology	Bereavement support group	5/purposive	Parents and spouse	Death
	Verwey et al. (2008)	Phenomenology	Hospital, pediatric unit	22/purposive	Parents	Conditions requiring medical or surgical interventions
	Mavundla et al. (2009)	Qualitative explorative descriptive	PHC facility	8/purposive, convenience	Parents, spouse, and siblings	Mental illness
	Mhaule et al. (2009)	Qualitative explorative descriptive	Rural community	12/purposive	Parents, adult child, siblings, and significant others	Mental illness
	Tshililo & Davhana-Maselesele (2009)	Qualitative descriptive	Home-based care	12/purposive	Mothers, spouses, and grandmothers	HIV/AIDS
	Majumdar & Mazaleni (2010)	Qualitative explorative descriptive	Community	9/not stated	Immediate female family	HIV/AIDS
		Phenomenology	Community	20/purposive	Parents and grandparents	Teenage pregnancies
	Roets et al. (2012)	Cross-sectional survey	Hospital, pediatric ICU	62/census	Mothers	Critical illness
	Sukumani et al. (2012)	Phenomenology	Home-based care	13/purposive	Parents, adult children, and grandmother	Tuberculosis
	Bejane et al. (2013)	Qualitative descriptive	PHC facility	8/purposive	Parents, aunts, and grandparents	HIV/AIDS
	Van Wijk et al. (2014)	Phenomenology	Health care center for rape victims	9/purposive	Male intimate partners	Rape
	Mokgothu et al. (2015)	Qualitative explorative descriptive	Hospital, mental health ward	9/purposive	Immediate family	Mental illness
	Botes & Langle (2016)	Quantitative, design not stated	Hospital, emergency	100/convenience	Parents, spouses, partners, adult children, siblings, and significant others	Injured
	Kisorio & Langley (2016)	Qualitative explorative descriptive	Hospital, ICU	17/purposive	Parent, spouse, adult child, sibling, and niece	End of life
	Oyegbile and Brysiewicz (2017a)	Cross-sectional survey	Hospital, ICU	162/census	Blood relatives, significant others	Critical illness
	Oyegbile and Brysiewicz (2017b)	Grounded theory	Hospital, ICU	9/convenience and theoretical	Parents, spouse, adult child and sibling	Critical illness
	Direng (2017)	Cross-sectional survey	Hospital, ICU	100/purposive	Parents, spouse, adult children, sibling, and significant other	Critical illness
	Tlhowe et al. (2017)	Phenomenology	Hospital, OPD	15/purposive	Parents and siblings	Mental illness
	Chironda and Bhengu (2018))	Qualitative case study	Hospital, renal unit	6/purposive	Spouse, adult child, parents, and sibling	Chronic Kidney disease
	Kay et al. (2018)	Qualitative explorative descriptive	Not stated	8/purposive	Parents, spouse, adult child, and uncle	Mental illness
	Potgieter & Maree (2018)	Qualitative descriptive	Hospital, cancer care center	11/purposive	Spouses and adult children	Cancer
	Maree et al. (2018)	Qualitative descriptive	Hospital, not stated	20/purposive	Immediate family	Cancer
	Kahonde et al. (2019)	Grounded theory	Community	25/convenience and snowball	Parents, aunt, and uncle	Intellectual disability
	Emmamally and Brysiewicz (2019)	Survey	Hospital, ED	353/purposive	Parents, spouse, grandparent, siblings, guardian, and significant others	Emergency illnesses
	Temane et al. (2019)	Phenomenology	Hospital, mental health institution	9/purposive	Couples	Mental illness
	Emmamally et al. (2020)	Qualitative descriptive	Hospital, ED	6/purposive	Spouses and adult children	Emergency illnesses
	Hlungwani et al. (2020)	Qualitative explorative descriptive	Hospital, mental health ward	8/purposive	Parents	Substance abuse
	Meintjes & Nolte (2015)	Qualitative case study	PHC facility	10/purposive	Parents	Atopic eczema
	Mooi & Ncama (2020)	Qualitative descriptive	Home-based care	4/purposive	Immediate family	Chronic critical-related malnutrition
	Mothwa et al. (2020)	Qualitative descriptive	Hospital, mental health ward	9/purposive	Parents and siblings	Mental illness
Swaziland	McCreary et al. (2004)	Qualitative explorative	Home-based care	21/convenience	Spouses, adult children, relatives, and neighbors	HIV/AIDS
West African region (single-country studies) (*n* = 12, 14.1**%)**						
**Ghana (*n* = 4, 4.7**%)**	Aziato & Adejumo (2014)	Qualitative explorative	Hospital, surgical ward	12/purposive	Parents, spouses, fiancé, and adult children	Surgical procedure
	Adama et al. (2018)	Phenomenology	Home-based care	30/purposive	Parents	Premature neonates
	Ohene et al. (2019)	Qualitative descriptive	Hospital, pediatric ward	19/theoretical	Parents	Road traffic accidents
	Kusi et al. (2020)	Phenomenology	Hospital, oncology unit	15/purposive	Mother, spouse, adult child, siblings, and friend	Cancer
**Nigeria** **(*n* = 8, 9.4%)**	Fatoye et al. (2006)	Cross-sectional survey	Hospital, medical ward	103/purposive	Spouse, adult children, relatives, and friends	Stroke
	Akpan-Idiok & Anarado (2014)	Cross-sectional survey	Hospital, OPD oncology	210/purposive	Spouses, partners, and parents	Cancer
	Achema & Ncama (2016)	Grounded theory	Hospital, pediatric unit	8/purposive	Parents	HIV/AIDS
	Oyegbile and Brysiewicz (2017a)	Mixed-method	Hospital/renal unit	96 and 15/purposive	Parents, siblings, and grandmothers	End-stage renal disease
	Oyegbile and Brysiewicz (2017b)	Qualitative descriptive	Hospital/renal unit	15/purposive	Parents, spouses, siblings, and adult child	End-stage renal disease
	Faronbi (2018)	Cross-sectional survey	Community	325/purposive	Parents, spouse & in-laws	Chronic diseases-
	Gabriel & Mayers (2019)	Quasi experimental	Hospital, OPD	108/convenience	Parent, spouse, child, sibling, and friend	Cancer
	Falade-Fatila & Adebayo (2020)	Cross-sectional survey	Community	367/multistage sampling	Male partners	Pregnancy care
**East and West African regions (multicountry primary study) (*n* = 1, 1.2%)**						
**Nigeria, Uganda, Zimbabwe**	Adejoh et al. (2021)	Qualitative descriptive	Hospital, palliative care providers	48/purposive	Spouses, parent, children, and siblings	Cancer
Central, east, west, and southern regions* (multicountry secondary studies) (*n* = 4, 4.7%)						
**Botswana **Malawi** **South Africa **Swaziland (Eswatini)** **Uganda**	Mclnerney and Brysiewicz (2009)	Systematic review	Desktop review	14 articles/not stated	Spouses, adult children, female relatives, and neighbors	HIV/AIDS
Cameroon, **Kenya, Malawi, Rwanda, Tanzania, Uganda, Zambia	Auvinen et al. (2013)	An integrative review	Desktop review	18 articles/Not stated	Males	Preventing Mother-to child transmission
**Ethiopia, Malawi,Mozambique, Nigeria, Senegal** **Tanzania, Uganda**	Nkwonta & Messias (2019)	Scoping review	Desktop review	18 articles/not stated	Males	Reproductive health interventions
**Botswana, **Kenya, **Namibia, **South Africa, Swaziland, Tanzania	Ntsayagae et al. (2019)	Meta synthesis	Desktop review	10 articles/purposive	Parents, spouses, grandparents, siblings, nephew, and nieces.	Mental illness

*Note.* PHC = primary health care; ICU = intensive care unit; NICU = neonatal intensive care unit; OPD = outpatient department.

WHO Afro-regions (*n* = 4) and included countries (*n* = 47). Context of single-study family nursing research in bold. *Contexts included in multicountry research.

** Middle-income countries

**Central African region**: (Angola, *Cameroon, Central African Republic, Chad, Congo Republic—Brazzaville, **Democratic Republic of Congo**, Equatorial Guinea, Gabon, São Tomé & Principe), **East African region**: (Burundi, Comoros, Djibouti, *Ethiopia, Eritrea, *Kenya, Madagascar, *Malawi, Mauritius, **Mozambique**, Réunion, *Rwanda, Seychelles, Somalia, Somaliland, *Tanzania, *Uganda, *Zambia, and *Zimbabwe**), Southern African region**: (*Botswana, Lesotho, *Namibia, *South Africa, *Swaziland), West African region: (Benin, Burkina Faso, Cape Verde, Côte D'Ivoire, Gambia, **Ghana**, Guinea, Guinea-Bissau, Liberia, Mali, Mauritania, Niger, *Nigeria, *Senegal, Sierra Leone, and Togo).

### Analysis of Evidence

**Figure 2.**
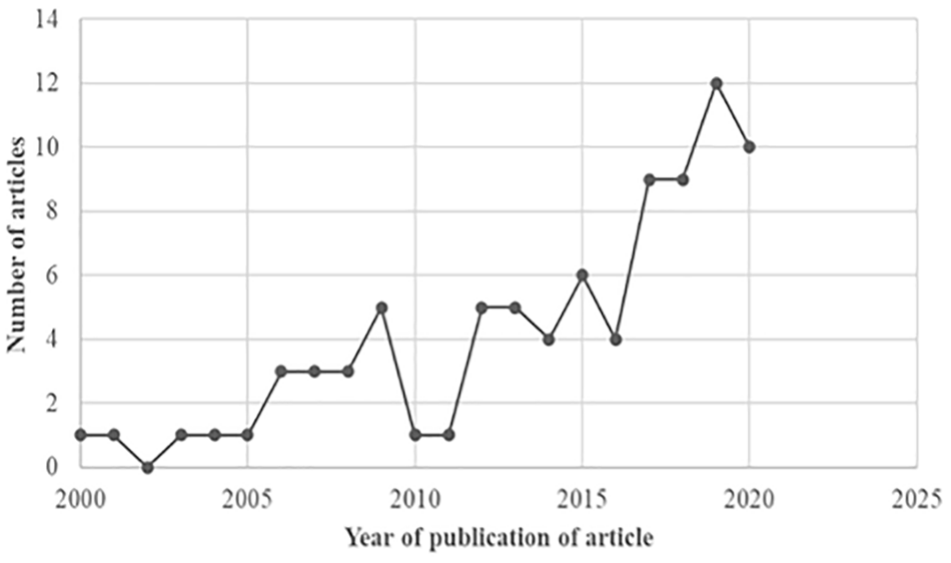
Publication Trends of Family Nursing Research in the WHO Afro-Region (n = 85)

**Figure 3.**
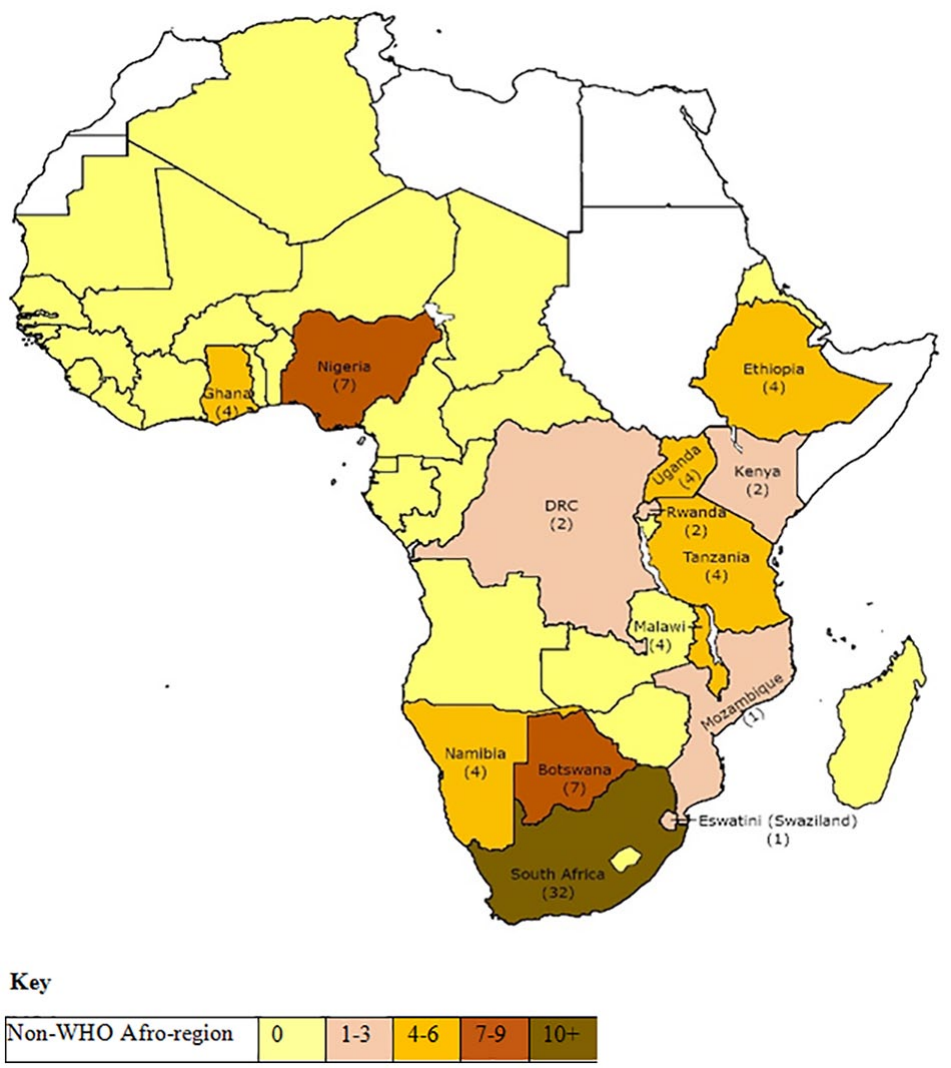
Number of Single-Country Studies in WHO Afro-Region States

**Figure 4.**
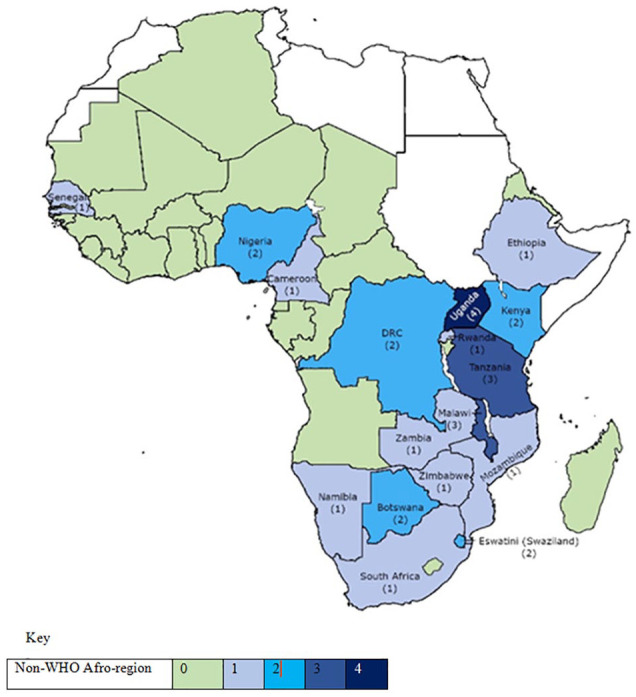
Number of Multicountry Studies in WHO Afro-Region States

The evidence was analyzed and represented through graphs (Figure 2), maps (Figures 3 and 4), and a synthesis table (Table 3). First, frequency counts of the number of articles published per annum from 2000 to 2020 identified family nursing research’s distribution and publication trends, specific countries, research designs/approaches used, and family concepts explored in the studies. After that, a descriptive summary presents a narrative of the results as they align with the five review questions. The scoping review followed the JBI methodology, hence it does not require appraising the methodological quality of the studies (Khalil et al., 2020; Peters et al., 2015).

## Results

### Search Results

The PRISMA-ScR flowchart (Figure 1) shows that the initial search revealed a total of 573 articles and ultimately resulted in 85 articles meeting the criteria for inclusion (Table 3).

### Publication Trends of Family Nursing Research in the WHO Afro-Region

Figure 2 depicts the number of articles published in English in the WHO Afro-region from 2000 to 2020. The year 2019 had the most publications (*n =* 12) and 2002 lacked any studies or reports on family nursing research. The identified articles were published in 36 different journals (*n =* 81) and gray literature through theses and dissertations (*n =* 4). A South African nursing journal, *Curationi*s (*n* = 14), was the journal most represented, while the majority of authors (*n =* 64) were affiliated with an academic institution. Psychologists, social workers, public health specialists, and medical doctors were other health care professionals involved in collaboration with family nurse researchers within and outside African academic institutions (United States, Finland, Australia, and Sweden).

### Distribution of Family Nursing Research

Single-country research involving families emanated from only 32% (*n* = 15) of the 47 WHO Afro-region member states, with the highest number of publications from Southern African-based studies (*n =* 44, 51.8%). The majority of these were from South Africa (*n* = 32, 37.6%), and the lowest from the Central African region (*n* = 2, 2.4%) (Figure 3; Table 3). The North African countries were not represented because they do not belong to the WHO Afro-region member states, and none of the island countries (Comores, Madagascar, Mauritius, and Seychelles) showed evidence of family-focused nursing research (Figure 3). Five studies involved a range of multicountries and showed the greatest involvement of Uganda (*n* = 4), followed by Malawi (*n* = 3) and Tanzania (*n* = 3) (see Figure 4; Table 3).

Using the classification of the Organisation for Economic Cooperation and Development (OECD), middle-income countries, namely, Botswana, Ghana, Namibia, Kenya, and South Africa contributed more than half of the studies (*n* = 48, 56.5%). Moreover, the Southern Africa region (mainly South Africa) offers doctoral programs that accommodate students from within Central, Eastern, and Western WHO Afro-member states. A difference was noted between the focus of the research settings used by the non-African authors, as primarily end of life care (hospices and home), compared with African authors whose studies were more often located within hospital settings.

### Types of Designs (Methodology) for Family Nursing Research

Predominantly, the selected articles employed qualitative methodologies (*n =* 59) over quantitative (*n* = 20), with fewer reviews (*n* = 5), and mixed-methods studies (*n* = 1). The most common study designs noted for qualitative studies was descriptive (*n* = 21), followed by phenomenology (*n* = 18), explorative descriptive (*n* = 11), grounded theory (*n* = 4), ethnography (*n* = 3), and two case studies. The most employed quantitative design was a descriptive cross-sectional survey, accounting for 15 of the 20 quantitative studies, with intervention studies (quasi-experimental) least represented (*n* = 2). Five reviews (scoping, integrative, narrative, qualitative meta-synthesis, and systematic) synthesized evidence for family-focused nursing (Table 3). Many studies (*n =* 48) were carried out in different departments of hospital settings with others including the community (*n* = 11), primary health care facilities (*n* = 10), and home-based care contexts (*n* = 9) (Table 3).

### Family Member(s) Involved and Focus Health Conditions

The majority of studies (*n* = 80) included a definition of family (who makes up the family) to include parents, legal guardian or next of kin, maternal or paternal relatives, neighbors, close friends, and significant others; however, the conceptualization of family (what is family) was not addressed. In line with the adopted definition of family in this review, members involved were predominantly parents, spouses, and adult children. Additional members included siblings, grandparents, relatives (unspecified), neighbors, and friends. The families were caring for members with the following focus conditions: HIV/AIDS (*n* = 14), mental illness (*n* = 12), cancers (*n* = 8), hospitalized critical illnesses (*n* = 8), and kidney diseases (*n* = 4) (Table 3).

### Family Nursing Research Focus

A significant number of studies (*n* = 53) focused on family experiences. Other studies focused on family needs (*n* = 7), family support, burden, and stress showing similar numbers (*n* = 6), and family engagement (*n* = 5). Family caregiving and family-centered nursing were the words commonly used to describe the type of care nurses offered to families.

## Discussion

The scoping review answered the five review questions, allowing the reviewers to synthesize and map the available literature sources (*n* = 85) on family-focused nursing research for the 46 WHO Afro-region member states (WHO, 2021) for the period 2000 to 2020. Conducting the scoping review proved challenging as evidence from the WHO Afro-regions showed variability in the definition of family.

### Publication Trends, Country Distribution, and Research Designs

In comparison to high-income countries, where an increase in publications on family-focused research began in the 1980s (Bell, 2019; Hanson, 2005), this review revealed that the WHO Afro-regions started at a slower pace. However, through increasing publication trends (2006-2020) there appears to be an increased interest in family nursing especially within the middle-income countries in the WHO Afro-region (South Africa and Kenya). The increase in African family nursing research holds specific relevance as the top 10 countries involving family nursing studies are ranked as United States, Brazil, Australia, Canada, Sweden, England, Japan, Finland, Germany, and the Netherlands (Huang et al., 2021); thus, some of the evidence-based practice produced may not be applicable or transferable to African countries (Sun, 2015).

The availability of research capacity, coupled with the development of research centers within specific Southern and Eastern African regions has served to attract funders (Morel et al., 2018), hence higher research outputs from these regions. The Southern African region, especially South Africa, showed accelerated growth from 2012 to 2020. Despite the evidence of growth, more than half (*N* = 31) of the countries within the WHO Afro-region do not have any family-focused nursing research outputs.

While there are limited family-focused publications within the WHO Afro-region, it is important to highlight the evidence of national, regional, and international multidisciplinary research collaborations (Adejoh et al., 2021; Aga et al., 2009; Fatoye et al., 2006; Hlungwani et al., 2020; Kululanga et al., 2012; McCreary et al., 2004; Musabirema et al., 2015; Nkosi et al., 2006; Nkwonta & Messias, 2019; Ntsayagae et al., 2019; Okumu et al., 2017; Outwater et al., 2012). However, despite the multidisciplinary involvement, compared with other health care professionals, nurses are often excluded and their voices are not considered in research (Holmes et al., 2020; Maree et al., 2017) despite being the front-line workers who deliver care. The observations of Holmes et al. (2020) and Maree et al. (2017) appear to hold true for family-focused research as evidenced by a significant number of articles being excluded from the review (*n* = 49) as they did not include a nurse author. Yet the nursing discipline is considered best suited to leading research of families that further informs policy for the health and well-being of families (Feetham, 2018).

In the clinical area, the absence of a skilled clinical research scholar (Conradie et al., 2018) and the shortage of nurses and midwives across the WHO Afro-regions (Sun, 2015) restricted research. Nonetheless, as the nurse might be less noticeable in multidisciplinary research, the majority of published research originated from academic institutions where nurse researchers are usually concentrated.

The recent external academic funding support for Masters and PhD students in Africa (Morel et al., 2018) may be the reason for increased research outputs from academic settings. However, Maree et al. (2017) have highlighted the difficulties which have arisen due to the shortage of knowledgeable faculty to supervise postgraduate students. Other authors have also provided reasons for a completed thesis/dissertation remaining as unpublished research due to limited availability of academics and students to develop a manuscript, the high rate of rejection by international journals regarding research from Africa, as well as the financial constraints in meeting publication costs (Bickton et al., 2019; Conradie et al., 2018).

Much of the included research was descriptive in nature, using a qualitative approach, similar to the findings of family nursing research reviews in high-income countries (Saveman, 2010; Østergaard & Wagner, 2014). A qualitative approach is predominant in nursing research (Doyle et al., 2020; Ganong, 2011), with the under-utilization of mixed-methods approach in family-focused nursing research not confined to Africa (Oyegbile & Brysiewicz, 2017a), suggesting nurse researchers’ limited experience in utilizing this methodology (Younas et al., 2019). Similarly, family interventions (Gabriel & Mayers, 2019; Shimpuku et al., 2018) are scarce in the WHO Afro-region. However, researchers are showing a growing interest in family nursing interventions, perhaps due to the highlighted importance globally of implementation research (Cassidy et al., 2021).

### Family Member(s) Involved and Focus Conditions

The current scoping review explored who was defined in the research as family, in keeping with international criteria for research of families (Feetham, 2018). The family members included in the studies were parents, siblings, adult children, grandparents, other relatives (aunts and uncles), friends, and neighbors. Makiwane and Kaunda (2018) identified the aforementioned family members as the ones who make up families in most African countries. The inclusion of “neighbor” and “friend” matches the definition of family in the current scoping review, thus showing variations in the progressive alteration of traditional African family structures.

Western conceptualizations of family as expressed in a review of Danish family nursing research of family being bound by legal, biological, adoptive, and/or martial ties (Østergaard & Wagner, 2014), can prove to be restrictive in the current African communities (Erlingsson & Brysiewicz, 2015). While the majority of Danish family nursing research was carried out in pediatrics (Østergaard & Wagner, 2014), nursing research involving families in WHO Afro-region states predominantly involved families of adult patients/clients.

The focus conditions of the studies included in the review featured chronic communicable (HIV/AIDS) and noncommunicable diseases (mental health, cancer, and kidney disease) posing a major burden in the WHO Afro-region (Bigna & Noubiap, 2019; Ellapen et al., 2021; Juma et al., 2018; Kaze et al., 2018). It is also worth noting that family-focused research appears to be influenced by patterns of foreign aid in African countries. For instance, the focus on HIV/AIDS and cancer is possibly due to the high influx of donor funding over the past years (van de Ruit, 2020). Furthermore, the inclusion of mental health in the 2015 Sustainable Development Goals (SDGs) (Docrat et al., 2019) might explain the growth of studies on families with members exhibiting mental health problems. Cancer and critical care nursing are well-established specialties in Africa (Maree et al., 2017), thus explaining research in these areas.

### Family Nursing Research Focus

There was a greater focus on family experiences of caring for various acute or chronic conditions within the different health care settings and communities than on family needs, support, burden, stress, and engagement. Not unique to the context of the WHO Afro-region, but similar to findings from a Swedish study (Saveman, 2010), the majority of African families experienced fear, physical, emotional, spiritual anguish, pain, loss of control, sorrow, confusion, and despair coupled with alterations in daily activities of living as they cared for their ill family members.

The African caregivers were not paid nor compensated in any way, as the caregiver perceived this role as a cultural obligation. Again, it is essential to look after the health care needs of an ill family member, with this role continuing into the health care facilities because of insufficient nurses in African countries (Nkengasong et al., 2021). Simultaneously, a financial obligation follows with possible economic burdens as the family is often expected to visit the hospital daily, as well as purchase medical supplies before their loved one can be treated (Aga et al., 2009; Chironda & Bhengu, 2018; Lindsey et al., 2003; Oyegbile & Brysiewicz, 2017b). Health care professionals and other family members endeavor to provide social, psychological, and emotional support (Adama et al., 2018; Direng, 2017; Emmamally & Brysiewicz, 2019; Emmamally et al., 2020; Letlola-Motana, 2007). However, financial support appears less possible due to the predominance of low-income countries within the WHO Afro-region.

Family experiences of changes in needs (Brysiewicz & Chipps, 2017, Hamukwaya, 2019; Meleis, 2010; Mooi & Ncama, 2020; Munyiginya & Brysiewicz, 2014; Ramnath, 2007) were mostly centered on families with loved ones in intensive care unit (ICU). Similarly, Swedish families with admitted family members in ICU felt that to promote the recovery process, it was necessary to stay close to the ICU bedside and, most importantly, there was a need for support from each other, even beyond discharge (Saveman, 2010). While Swedish family nursing research identified hope, a positive attitude toward life, and attempting to live a normal life as a way to cope with the illness of a family member (Saveman, 2010), how families adjust and cope throughout the lifespan after the illness of a family member is not well researched in the WHO Afro-region.

Moreover, the increasing burden of chronic illnesses, home-based palliative care, and hospitalization of end of life individuals have been noted, with only a few studies (Fatoye et al., 2006; Gabriel & Mayers, 2019; Kisorio & Langley, 2016; McInerney & Brysiewicz, 2009; Oyegbile & Brysiewicz, 2017a; Philips & Lazenby, 2013) focused on these areas. Yet, more studies on home-based palliative care emanated from a family nursing research review done in Sweden over a decade ago (Saveman, 2010). Similarly, despite Africa’s turbulent socio-economic political history, with an incumbent high mortality rate due to injuries and diseases, including the current Covid-19 pandemic (Iheonu et al., 2019; Lone & Ahmad, 2020), experiences involving family loss (Brysiewicz, 2008; Davhana-Maselesele, 2005; Outwater et al., 2012) are not well explored, evidenced by last published study in 2012 (Outwater).

In addition, compared with family nursing research in Finland, where family violence studies rank high as a focus area (Åstedt-Kurki, 2010), and interestingly counter to sub-Saharan Africa’s (SSA) high violence prevalence (Cools et al., 2005), there were only a scarce number of studies on family violence and abuse (Mugoya et al., 2020; Van Wijk et al., 2014). However, on the opposite side of the pendulum of violence, the classic African philosophy of *Ubuntu* encompassing compassion and humanity rooted in their beliefs, cultural influences and relationships, was only explored through three studies (Kahonde et al., 2019; Leuning et al., 2000; Temane et al., 2019). Similarly, Ganong (2011) reported limited family nursing research studies on cultural issues that affected families at a global level. It is interesting to note studies that emphasized the concept of family engagement (Falade-Fatila & Adebayo, 2020; Jaana et al., 2013; Nkwonta & Messias, 2019; Shimpuku et al., 2018) involved males in various programs that pertained to antenatal and reproductive care, thus indicating a growing interest by black African men in women’s health.

### Strengths and Limitations

To our knowledge, this is the first attempt to map the evidence of family-focused nursing research in the WHO Afro-regions. Three reviewers selected articles independently, which eliminated possible selection bias. Furthermore, gray literature sources in the form of completed academic theses and dissertations were examined to add to the evidence that was relevant to this scoping review.

Families have been part of nursing care since Florence Nightingale and nurse researchers have access to the scholarship of family science across disciplines. Hence, the use of family experiences terminology was an appropriate approach among African family nurse researchers. The articles described the definition and structure of the family in Africa per se, as opposed to reliance on research from non-African countries.

The review had some limitations in that, across all the published studies, there does not appear to be a common understanding of the concept of family-focused nursing. Therefore, it was challenging to define search terms that were sensitive to the central concept of family nursing research when “family focused nursing” is not an accepted nor well-understood term in Africa.

Family nursing is not a recognized specialty in the WHO Afro-region, hence, the unfamiliarity of using family nursing constructs among the nurse researchers. It then becomes challenging for the reviewers to identify family constructs within the research.

Only countries in the WHO Afro-region were included thus eliminating the North African countries belonging to the Eastern Mediterranean Region.

There have only been four similar national reviews focused on the science of family nursing (Denmark, Finland, Sweden, and Japan) (Østergaard & Wagner, 2014; Åstedt-Kurki, 2010; Ganong, 2011; Saveman, 2010), therefore, the reviewers had limited information against which to compare and contrast the current findings.

Only articles written in English were selected, meaning any publications in Portuguese, Arabic, Amharic, French, and local African languages could have been excluded. Articles without a nurse as either an author or co-author were excluded, and it is possible that some studies in which nurses participated were excluded.

Patient and public involvement in the research process of the extracted studies was not explored and warrants further investigation as this has been shown to improve the quality and end results of research (Tomlinson et al., 2019).

### Implications for Family Nursing Practice

Despite the development of family-focused nursing at a global level, it is not formally recognized in the WHO Afro-regions. Hence, there is a need to introduce family nursing in nursing curricula in Africa. This could prepare and equip nurses with additional knowledge on how to engage with families and promote the implementation of family-focused nursing research, thus bridging the gap between research and practice. Many of the countries in the WHO Afro-regions, where there was no evidence of family-focused nursing research, might have perceived other health needs as priorities, offering a unique opportunity to concurrently address health needs and introduce family-focused nursing.

### Implications for Research About Families

There is a paucity of literature on family-focused nursing research in the WHO Afro-region and the available research is primarily qualitative and descriptive in nature. However, the limited use of quantitative, mixed-methods, and specifically grounded theory, ethnographic, and case study qualitative research designs, suggests there is a larger scope needed in future research to build evidenced family nursing theory relevant to the African context. In addition, family nurse researchers need to focus on the impact of family nursing or family care and make use of more complex and sophisticated methodologies to create evidence-based practice for patients and their families within Africa.

In addition, findings from the current review may be used to strengthen research agendas related to family-focused nursing research by placing more emphasis on transforming the African nursing curricula to include the specialty of family nursing to facilitate the development of family nurse researchers in both academic and clinical settings. The review revealed that the majority of the WHO Afro-region member states hold the opportunity for the beginnings of research in family-focused nursing.

## Conclusion

Despite the recent increase in family-focused nursing research in the WHO Afro-regions, further qualitative research including more complex methodologies and interventions, are still required to extend family nursing research beyond the identified countries. Inclusion of family nursing into the nursing curricula will help to position the science and practice in the teachings of family-focused nursing education, policy development, and research and help build an evidence-based family nursing practice for the WHO Afro-region.
